# Proteoform Analysis of Matrix Metalloproteinase-9/Gelatinase B and Discovery of Its Citrullination in Rheumatoid Arthritis Synovial Fluids

**DOI:** 10.3389/fimmu.2021.763832

**Published:** 2021-11-29

**Authors:** Bernard Grillet, Karen Yu, Estefania Ugarte-Berzal, Rik Janssens, Rafaela Vaz Sousa Pereira, Lise Boon, Erik Martens, Nele Berghmans, Isabelle Ronsse, Ilse Van Aelst, Pierre Fiten, René Conings, Jennifer Vandooren, Patrick Verschueren, Jo Van Damme, Paul Proost, Ghislain Opdenakker

**Affiliations:** ^1^ Laboratory of Immunobiology, Rega Institute for Medical Research, Department of Microbiology, Immunology and Transplantation, KU Leuven, Leuven, Belgium; ^2^ Laboratory of Molecular Immunology, Rega Institute for Medical Research, Department of Microbiology, Immunology and Transplantation, KU Leuven, Leuven, Belgium; ^3^ Skeletal Biology and Engineering Research Center, Department of Developmental and Regenerative Medicine, University Hospitals Leuven, Leuven, Belgium

**Keywords:** matrix metalloproteinase, arthritis, proteoform, proteolysis, citrullination, synovial fluid, post-translational modification

## Abstract

**Objectives:**

To explore posttranslational modifications (PTMs), including proteolytic activation, multimerization, complex formation and citrullination of gelatinases, in particular of gelatinase B/MMP-9, and to detect in gelatin-Sepharose affinity-purified synovial fluids, the presence of specific MMP proteoforms in relation to arthritis.

**Methods:**

Latent, activated, complexed and truncated gelatinase-A/MMP-2 and gelatinase B/MMP-9 proteoforms were detected with the use of zymography analysis to compare specific levels, with substrate conversion assays, to test net proteolytic activities and by Western blot analysis to decipher truncation variants. Citrullination was detected with enhanced sensitivity, by the use of a new monoclonal antibody against modified citrullines.

**Results:**

All MMP-9 and MMP-2 proteoforms were identified in archival synovial fluids with the use of zymography analysis and the levels of MMP-9 *versus* MMP-2 were studied in various arthritic diseases, including rheumatoid arthritis (RA). Secondly, we resolved misinterpretations of MMP-9 levels *versus* proteolytic activities. Thirdly, a citrullinated, truncated proteoform of MMP-9 was discovered in archival RA synovial fluid samples and its presence was corroborated as citrullinated hemopexin-less MMP-9 in a small prospective RA sample cohort.

**Conclusion:**

Synovial fluids from rheumatoid arthritis contain high levels of MMP-9, including its truncated and citrullinated proteoform. The combination of MMP-9 as analyte and its PTM by citrullination could be of clinical interest, especially in the field of arthritic diseases.

## Introduction

Rheumatology research has witnessed leaps of progress by the combination of basic and clinical research. This resulted in the therapeutic blockade of tumour necrosis factor (TNF) signalling in RA. This breakthrough anticipated and came without sophisticated high-end single cell RNA-sequencing analysis (1). At the diagnostic level, the discovery of antibodies against citrullinated peptides in RA was another breakthrough. Citrulline is an essential constituent of antigenic determinants for RA-specific autoantibodies (2). The detection of circulating antibody titers against citrullinated (cyclic) peptides (CCP), e.g. anti-citrullinated peptide antibodies (ACPA) is used today for differential diagnosis, therapeutic follow-up and prognosis of patients with various forms of arthritis (3, 4).

Matrix metalloproteinases (MMPs) have also been extensively studied in arthritis. Pioneering studies also date back to 1987 when Okada and colleagues described MMP-1/fibroblast collagenase, MMP-2/gelatinase A and, most significantly, MMP-3/stromelysin-1 in association with arthritic diseases. MMP-3, together with MMP-1 and MMP-2, is able to destroy cartilage (5). In an animal model of antibody-induced arthritis, MMP-2-deficient mice had increased, whereas MMP-9-deficient mice had decreased arthritis scores, indicating a protective role for MMP-2 and detrimental effects of MMP-9 (6). A role for the membrane-type (MT)1-MMP/MMP-14 has also been described. Inhibition of MT1-MMP in collagen-induced arthritis in mice resulted in reduced disease progression in synergy with anti-TNF treatment (7). Aside increased levels of ACPA, the levels of MMPs, in particular of MMP-3, are increased in sera and synovial fluids of arthritic patients (8) and their detection in plasma/serum or synovial fluid may be used for diagnostic purposes of, respectively, systemic and local disease. The direct functions of MMPs in joint destructions, by collagenolysis and degradation of proteoglycans, have been recognized for a long time (9). The indirect effects of MMPs, leading to the understanding of the autoimmune processes took longer to be accepted and went from the identification of neutrophil MMP-9/gelatinase B (10, 11), *via* the discovery of glycosylated remnant epitopes of type II collagen (12) to studies evaluating the therapeutic effects of MMP inhibitors (13, 14) in autoimmune diseases (15).

We here provide details about proteoforms of MMP-9 and MMP-2 in human synovial fluids and describe how citrullination of MMP-9 was discovered in samples from a large patient cohort. Our data were enhanced thanks to new insights (16) and were reinforced by analysis of samples from a small prospective translational study.

## Materials and Methods

### Proteins, Antibodies and Inhibitors

Recombinant full-length human proMMP-9 and a mutant lacking the hemopexin and the *O*-glycosylated domains were used as markers in zymography analysis (17). Full length and deletion mutated MMP-9 proteins were produced by recombinant expression in Sf-9 cells and purified as described previously (16, 18). For the detection of human MMP-9 by Western blot analysis, 3 µg/mL goat anti-MMP-9 IgG1 antibody (R&D Systems) and a 1/10 000 dilution of peroxidase-labelled horse anti-goat antibody were used. For the detection of the hemopexin domain of MMP-9, we used rabbit anti-PEX9 IgG antibody diluted 1/1000 (kindly provided by Prof. Angeles Garcia Pardo, Consejo Superior de Investigaciones Científicas, Madrid, Spain (19)) and 1/2500 diluted peroxidase-labelled donkey anti-rabbit antibody. For the detection of citrullinated proteins in archival samples, a 1/1000 dilution of polyclonal anti-modified citrulline antibody (#17-347, Millipore) was used. To detect chemically modified citrulline residues, a new monoclonal mouse antibody was produced in-house and used at a concentration of 3 µg/mL. In all Western Blots, a protein marker (Precision Dual Colour PLUS, BioRad) was run in parallel to indicate reference molecular weights. For indirect ELISAs, 1 µg/mL of the in-house produced monoclonal antibody was used as primary antibody and further probed with a peroxidase-labelled goat anti-mouse antibody (R&D Systems) diluted 1/10 000.

### Establishment of a New Monoclonal Antibody Against Modified Citrullines

BALB/c and C57BL/6 mice were immunized with chemically modified (*) citrulline peptides (PIECit*TYLK-NH_2_ and YAGCit*LLTK-NH2), coupled to either keyhole limpet hemocyanin (KLH) or ovalbumin. Different citrulline-containing peptides were created in the host laboratory *via* solid phase peptide synthesis, as described previously (20). Hybridoma cell lines were created using the ClonaCell®-HY kit (Stemcell Technologies) according to the manufacturer’s instructions. SP2/0 mouse myeloma cells were fused with purified splenocytes of immunized mice. Stable hybridomas were screened and selected on the basis of antibody reactivity to different synthetic citrullinated and control peptides that were coated on the plates at 100 ng/ml with the use of indirect ELISAs (20). Further validation was performed by Western Blot analysis and the use of synthetic citrullinated and non-citrullinated CXCL8 isoforms (21). Cell culture supernatants were filtered and antibodies were purified on Protein G affinity chromatography (GE Healthcare).

### Gelatin-Sepharose Purification

To purify MMP-9, samples were loaded in 50 mM Tris pH 7.4, 0.5 M NaCl, 10 mM EDTA, 15 mM CaCl_2_ on gelatin-Sepharose (GE Healthcare) micro-columns (Biorad). Samples were incubated to allow binding to gelatin and subsequently washed with 50 mM Tris pH 7.4, 1.5 M NaCl, 10 mM EDTA, 15 mM CaCl_2_. Bound proteins were eluted with reducing loading buffer [10 mM Tris, 1 mM EDTA, 50% (w/v) glycerol, 0.05% (w/v) bromophenol-blue, 10% 2-mercaptoethanol] (22).

### Immunoblot Analysis of Citrullinated Proteins After Chemical Modification

Prior to SDS-PAGE, proteins were denatured in reducing conditions and by heat incubation at 95°C. Proteins were separated in 16% or 4-20% gradient Tris-glycine gels and transferred to polyvinylidene difluoride (PVDF) membranes (Biorad). For citrulline detection, proteins were incubated with 0.25% 2,3-butanedione, 0.125% antipyrine; 0.0125% FeCl_3_, 0.25 M CH_3_COOH, 2.2 M H_2_SO_3_, 1.5 M H_3_PO_4_ for chemical modification of citrulline residues (23) immediately after protein transfer to PVDF membranes. After blocking with 5% BSA in TBS-Tween, the membranes were probed with the in-house generated antibody recognizing modified citrullines by the anti-modified citrulline (AMC)-method (23, 24). As secondary reactants, we used peroxidase-conjugated goat anti-mouse IgG. Detection was performed after addition of chemiluminescent substrate (Super Signal West Pico #34,080 and Femto #34,095, ThermoFisher, Waltham, MA, USA). Equal amounts of biological samples were used for comparisons and similar time exposures were used to semi-quantify chemiluminescent intensity signals.

### Gelatin Zymography Analysis

Samples of 1 µl crude synovial fluids were diluted as indicated (1/10, 1/100 or 1/200), analysed on 7.5% polyacrylamide gels containing 0.1% gelatin, as previously described (16). Trimeric and monomeric proMMP-9 and a specific deletion mutant of human proMMP-9 (lacking the *O*-glycosylated and the hemopexin domains) were included as molecular size markers and for standardization of the gelatin zymography method (17, 25).

### Gelatinase Activity Assay

Gelatinase activity in synovial fluid samples was measured by fluorescent-activated substrate conversion (FASC), a method that detects *in vitro* degradation of fluorescently labelled gelatin coated on polystyrene microparticles. This technology has been detailed elsewhere and may be executed on any simple flow cytometer (26).

### Interleukin-6 and Interleukin-8/CXCL8 Assays

The analysis of IL-6 was performed with a functional bio-assay on the basis of growth of an IL-6-dependent hybridoma cell line (27). IL-8/CXCL8 was measured with a radio-immunoassay as described (28).

### Patient Samples

Blood and synovial fluids of arthritis patients, both stable and during exacerbation, were collected after obtaining written informed consent of patients, in accordance with the Declaration of Helsinki and upon approval of the local Ethics Committee (ML1814 KU Leuven, Belgium). Synovial fluids were aspirated from the tibiofemoral joint of patients who suffered from clinical swelling and inflammation. The primary purpose for joint aspiration was therapeutic relief and diagnosis. Arthritis patients were classified according to the revised criteria of the American Rheumatism Association of 1987.

### Quantification, Image and Statistical Analysis

Graphs were generated and statistical analysis was performed using Prism (GraphPad). Western blot images were merged using Adobe Photoshop version 22.4.1. Normality was assessed with the use of the Shapiro-Wilk normality test; all data points tested were normally distributed. One-way ANOVA was used to calculate matrix metalloproteinases values in between different MMP-9 forms. All p values <0.05 were considered significant.

## Results

### Gelatinase Proteoforms in Synovial Fluid

**Figure 1:**
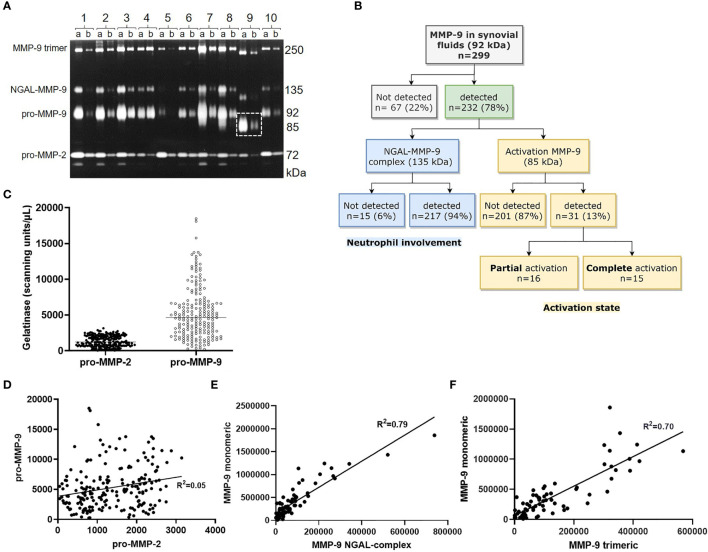
Synovial fluid analysis with the use of gelatin zymography. Diluted synovial fluids, 1/10 **(lanes a)** and 1/100 **(lanes a)**, were analysed by gelatin zymography. Sharp bands at 72 kDa represent pro-MMP-2, whereas unsharp 92 kDa bands correspond to monomeric pro-MMP-9 glycoforms. NGAL binds pro-MMP-9 and yields gelatinolysis around 135 kDa. Homotrimers migrate at approximately 250 kDa, whereas activated proteoforms (lane 9a and 9b) contain monomers at 85 kDa (dashed rectangle) **(A).** Flowchart with proteoform detection in a historical patient cohort **(B).** proMMP-9 levels vary considerably more compared to proMMP-2 **(C)** and appear unrelated (n=200, r^2 =^ 0.05) **(D)**. Monomeric MMP-9 levels correlate with amounts of NGAL-complexed MMP-9 (n=72, r^2 =^ 0.79; p<0.0001) **(E)** and with MMP-9 homotrimers (n=71, r^2 =^ 0.70; p<0.0001) **(F)**.

Before the instatement of the General Data Protection Regulation (GDPR) law in Europe (2016) and in full compliance with the Declaration of Helsinki, a considerable library of synovial fluid samples of patients with various forms of arthritis was assembled between 1990-1999. This patient cohort was analysed by gelatin zymography **(** **Figure 1A** **)** and used to define all gelatinase proteoforms as summarized in **Figure 1B**. All samples contained the pro-form of MMP-2/gelatinase A as a well-delineated protein of 72 kDa. MMP-2 was corroborated to be constitutively expressed. In contrast, monomeric pro-MMP-9 levels were highly variable and represented mixtures of various glycoforms, visualized as unsharp gelatinolysis zones around 92 kDa. Neutrophils produce, aside from pro-MMP-9, a 25 kDa neutrophil gelatinase B-associated lipocalin (NGAL). In addition to the NGAL monomer of 25 kDa, NGAL forms glycosylated homodimers and heterodimers, linked covalently to pro-MMP-9 within these cells (29, 30). Heterodimer detection may be used as a surrogate biomarker for the presence and activated state of neutrophils (31).

Together with pro-MMP-9, most cell types also co-produce the tissue inhibitor of metalloproteases-1 (TIMP-1) and circular homotrimers of MMP-9 (25, 32). Significantly, neutrophils are an exception and do not produce TIMP-1 (33). Aside from the detection of NGAL-MMP-9 complexes, we observed in all synovial fluids with detectable pro-MMP-9 monomers also the presence of pro-MMP-9 trimers **(** **Figure 1A** **)**. Finally, in a limited number of samples we detected activated forms of MMP-9 (*vide infra*). For the three proteoforms (monomeric forms, heteromeric complexes with NGAL and trimeric MMP-9) in arthritic synovial fluids, the proteolytically activated MMP-9 proteoforms migrated at correspondingly lower molecular weights **(** **Figure 1A**, lane 9), whereas most of the constitutive pro-MMP-2 remained at 72 kDa. Therefore, the analysis of MMP-9 levels by commonly used ELISAs in clinical studies is of limited value, because it yields single numbers for the ensemble of all MMP-9 proteoforms present in single samples. For these reasons, we preferred to rely on gelatin zymography analyses of individual proteoforms. Variability was illustrated by combining the absolute gelatinolysis data (by MMP-2 and MMP-9 monomers) from six consecutive patient cohort experiments. For all six cohorts, individually and pooled together, the levels of pro-MMP-2 varied relatively little in comparisons with the levels of pro-MMP-9 proteoforms. The distributions of absolute levels within the complete pooled sample set are demonstrated in **Figure 1C**.

The presence of synovial NGAL/MMP-9 complex suggested neutrophil involvement (*vide supra*). Correlations between synovial levels of interleukin-8 (IL-8/CXCL8), local neutrophil counts and absolute MMP-9 levels have been previously documented (12, 28). We therefore complemented these data with correlation analysis based on MMP-9/MMP-2 levels (B/A ratios) and systemic biomarkers of inflammation. The synovial B/A ratios did not correlate with systemic markers of inflammation, including erythrocyte sedimentation rates **(** **Supplementary Figure S1A** **)**, C-reactive protein levels **(** **Supplementary Figure S1B**) and absolute neutrophil counts in venous blood **(** **Supplementary Figure S1C** **)**.

### Gelatinase Activities in Synovial Fluid as Disease Parameter of Arthritis

We used gelatin zymography analysis to detect MMP-9 proteoforms. In 22% of the samples no MMP-9 was detected, whereas 217 (94%) of 232 MMP-9-positive samples contained monomeric pro-MMP-9 together with the presence of the NGAL complex (as a marker for the presence of neutrophils) and the pro-MMP-9 trimers **(** **Figure 1B** **)**. In 31 (13%) of the 232 MMP-9-positive samples, we observed proteolytic activation of monomeric pro-MMP-9 of 92 kDa to the 85 kDa activation proteoforms (see **Figure 1A**, lane 9), aside activation forms of the NGAL complex and the trimers. This activation was partial in 16 samples, as assessed by the presence of both intact monomers at 92 kDa and activated MMP-9 glycoforms around 85 kDa, whereas the proteolytic activation was complete in 15 of the 31 samples **(** **Figure 1B** **)**.

To detect net activities in synovial fluid, we relied on the fluorescent-activated substrate conversion (FASC) method. Upon activation, fluorescent gelatin will be cleaved off from microparticles and the resulting beads will yield proportionally less fluorescence (26). Of note, a disadvantage of this and many other activity tests is that these techniques yield total gelatinase activity, indiscriminative of whether executed by MMP-2, MMP-9 or other gelatinolytic enzymes in the samples. We analysed 254 synovial samples from patients, about 60% having RA, 12% having spondylarthritis and 3% having degenerative joint diseases. We detected net gelatinolytic activity in 60 of the 254 samples (24%). Despite the presence of MMP-2 or MMP-9 or both in all samples, as detected by gelatin zymography, 194 samples were devoid of net activity. Because MMP-2 is constitutively present in all samples and to allow comparisons between individual zymography gels, we used the MMP-9/MMP-2 ratio or B/A ratio as a parameter. Although the mean B/A ratio was higher in samples with net activity than in those without, this difference was not significant (6.3 *versus* 5.9, Student T-test t=0.473; p>0.5).

The 85 kDa proteoform, observed in 13% of the synovial fluid samples **(** **Figure 1B** **)** corresponded with activated MMP-9 and this was reinforced by correlation analysis with its net activities. 16 of 53 samples (30%) with net activity had the 85 kDa band, in comparison with 11 of 181 samples (6%) without net activity. As such, 16 of 27 samples with the 85 kDa proteoform of MMP-9, i.e. 59%, possessed net gelatinolytic activity. Since MMP-2 is a constitutive enzyme in synovial fluid, our data provided ground for a strong association of 85 kDa MMP-9 and net gelatinolytic activity (Chi-square = 24,1; p<0.001) and for assigning 85 kDa MMP-9 as the activated form yielding, together with the activated NGAL-complexed and the activated trimeric proteoforms, the net activities.

The zymolytic gelatinase B/A ratio in synovial fluid was not related to systemic parameters of inflammation in venous blood **(** **Supplementary Figure 1** **)**. We also compared associations of net gelatinase activities in synovial fluids with blood biomarkers of inflammation **(** **Table 1** **)**. FASC activities in synovial fluids were associated with increased erythrocyte sedimentation rates, levels of C-Reactive Protein but not with rheumatoid factor. In contrast, in synovial fluid, local neutrophil counts and IL-6 levels were related with net gelatinolytic activities, whereas IL-8/CXCL8 levels were not. No relations were found between the levels of pro-MMP-2 or pro-MMP-9 **(** **Figures 1C, D** **)** and of the net activities contained in these samples after FASC analysis (pro-MMP-2, n=40, R=-0.14, p=0.38) (pro-MMP-9, n=40, R=0.15, p=0.32), whereas levels of MMP-9 monomers, NGAL complexes and trimers were correlated **(** **Figures 1E, F** **)**. When the net activities in the samples with the 85 kDa activation form of MMP-9 were evaluated, significantly higher activities were observed in comparison with the samples with only the pro-MMP-9 proteoform. (Wilcoxon comparison: Z=3.22; p<0.002). The samples with net activity had also higher synovial neutrophil counts than those without net activity (13320 +/- 11750 cells per µl, *versus* 7000+/-4100 cells per µl: Student t-test t =2.08, p<0.05), whereas by gelatin zymography test the gelatinase B levels were comparable (B/A ratio 8.56 +/-14.08 *versus* 8.00 +/- 16.41: no significant difference).

**Table 1:** Correlation analysis between gelatinase activity and venous blood and synovial fluid markers of inflammation.

Blood	FASC-positive Mean (SD)	FASC-negative Mean (SD)	p-value
ESR (mm/h)	60.1 (34.7)	43.4 (31.0)	<0.001
CRP (mg/dl)	6.37 (5.36)	4.42 (4.56)	<0.05
Rheumatoid factor (IU/ml)	66.8 (124.9)	92.2 (359.6)	0.57
**Synovial fluid**			
IL-6 (log10 U/ml)	3.46 (0.72)	3.07 (0.78)	<0.01
IL-8/CXCL8 (ng/ml)	11.5 (12.5)	10.6 (11.4)	0.72
Neutrophils (x 1000/µl)	14.86 (8.46)	6.15 (5.40)	<0.01

ESR, erythrocyte sedimentation rate; CRP, C-reactive protein.

Six consecutive patient cohorts (n=254).

In conclusion, the detection by zymography analysis of the 85 kDa activation proteoform of MMP-9 was a complementary marker for net gelatinase activity. Net gelatinolytic activities, IL-6 levels and local neutrophil counts in synovial fluids were important local biomarkers, in line with systemic disease parameters of arthritis.

### RA Synovial Fluid Contains a Citrullinated Gelatinase B Proteoform

In order to detect citrullination of MMPs in synovial fluid, 4 ml samples of starting material and selected samples with the highest pro-MMP-9 levels were concentrated by affinity purification (22). We selected 5 samples from osteoarthritis (OA) and 5 from RA patients. In **Supplementary Figure 2**, we demonstrated a weak citrullination signal in some of these RA samples, whereas all 5 OA samples were devoid of such immunoreactivity (**Supplementary Figure 2A**). In particular, two RA samples yielded weak signals for a citrullinated protein band at 57 kDa (red arrows) and a smear between 57 kDa and 114 kDa, aside immunoreactive peptides migrating below 14 kDa. The assigned molecular size (57 kDa) of the faint bands was validated by calculating the relative mobility as indicated (**Supplementary Figure 2B**, red diamond). Recently, we demonstrated the existence of a hemopexin-less 57 kDa MMP-9 proteoform, generated by MMP-3 when human MMP-9 is hypercitrullinated (16). Furthermore, it is known that stimulated human neutrophils produce various aminoterminal fragments of MMP-9 with gelatin-binding properties and having molecular weights in the low size range around 10 kDa (10) and containing PAD accessible sites for citrullination (16). Therefore, we first improved citrulline detection.

### A New Monoclonal Antibody for Detection of Citrullines in Proteins

**Figure 2:**
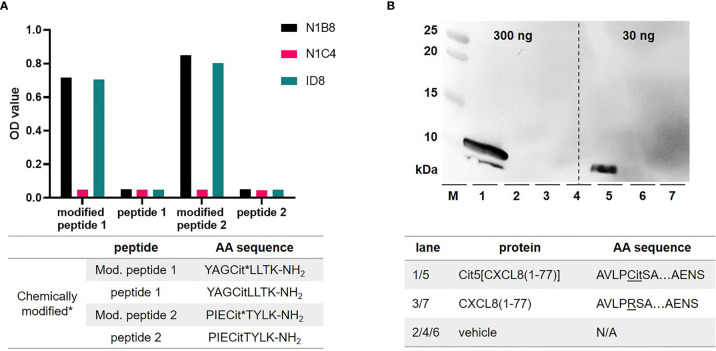
Production and validation of monoclonal anti-modified citrulline antibodies. Hybridoma clones, both generated from modified peptide 2, were screened with ELISAs, reactive towards different synthetic citrulline (Cit) containing peptides. These were chemically modified (* asterisk), and their unmodified isoforms served as negative controls **(A)**. Purified monoclonal antibodies were validated with in-house CXCL8 isoforms. By Western Blot analysis and a protein marker (M) we showed specific detection of citrullinated CXCL8 up to 30 ng and confirmed that the monoclonal antibodies did not cross-react with native CXCL8, which lacks citrulline **(B)**. Amino acid (AA) sequences of respective synthetic peptides are provided in corresponding tables below each panel. Not applicable (N/A).

Previously, we detected citrullination with polyclonal antibodies (20). We produced here monoclonal antibodies against modified citrulline with improved affinity and detection sensitivity in immunohistochemistry analysis. Initially, BALB/C and C57BL/6 mice were injected with two different citrulline peptides, namely modified peptide 1 and 2 (amino acid sequence provided in **Figure 2A**). Each modified peptide was coupled as immunogen to a carrier protein to create mouse hybridoma cell lines. Clone selection was performed *via* ELISA, that was designed to determine the specificity of the antibodies towards modified citrulline, and not the peptide backbone, which is a major pitfall in this process. BALB/C mice immunized with modified peptide 2 on the carrier provided clones with the highest signals in capturing modified citrullines. 48 different colonies originating from one hybridoma were screened (data not shown) and this led to the selection of N1B8 and ID8 monoclonal antibodies. These two antibodies were highly specific for modified peptidylcitrulline in ELISA **(** **Figure 2A** **)**. In forthcoming experiments, we used the N1B8 clone and further validated this monoclonal antibody *via* western blotting to determine its sensitivity and specificity toward citrullinated peptides. The sensitivity for detecting proteins with modified citrulline with this monoclonal antibody in western blot was in the nanogram-range. For quality controls we used CXCL8 proteins, which were chemically synthesized in our laboratory (21). Our results clearly indicated that the antibody did not recognize native CXCL8 and was only reactive to CXCL8 including a peptidylcitrulline residue **(** **Figure 2B** **)**.

### Citrullinated MMP-9 Proteoforms Are Detected in Arthritic Synovial Fluids

**Figure 3:**
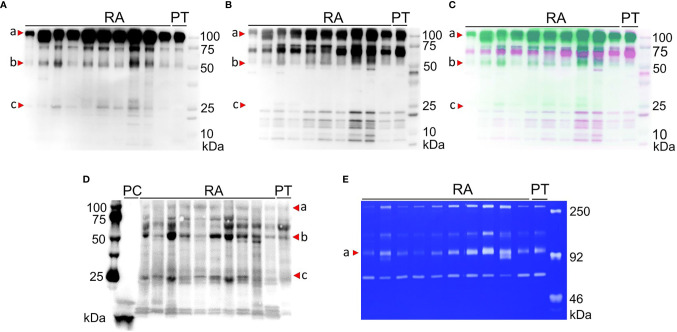
Citrullinated MMP-9 proteoforms are detected in arthritic synovial fluids. Western blots of synovial fluids of 10 rheumatoid arthritis (RA) and 1 post trauma (PT) patient were probed for MMP-9 **(A)** and its hemopexin domain **(B)**. False-colour overlay of image **(A)** (green) and **(B)** (purple) revealed uniquely green 57 kDa bands (b), thus representing hemopexin-less MMP-9, whereas degraded hemopexin fragments (c) appear purple **(C)**. We identified intact MMP-9 (a) and citrullinated MMP-9 proteoforms at 57 kDa (b) and 25 kDa (c) by modified citrulline detection. Citrullinated CXCL8 was positive control (PC) **(D)**. Zymographic analysis showed differential levels of MMP-2 and MMP-9 proteoforms in crude synovial fluids **(E)**.

Next, we analysed a prospective patient cohort of 10 RA patients and 1 post-trauma (PT) patient. We purified synovial fluid samples by gelatin-Sepharose affinity chromatography and subjected the bound peptides to Western blot analysis. Probing with a polyclonal antibody against MMP-9 showed the presence of intact MMP-9 (92 kDa, red arrowhead a) and proteolytic fragments at approximately 57 kDa (red arrowhead b) and below 25 kDa (red arrowhead c) in a number of samples **(** **Figure 3A** **)**. To identify the MMP-9 fragments, the blot was stripped and reprobed with an antibody that detects the hemopexin domain of MMP-9 (PEX9). As a result, immune reactivity was concentrated at 92 kDa, marking intact proMMP-9 with the hemopexin domain. In contrast, reactive bands were barely visible at 57 kDa **(** **Figure 3B** **),** in line with a hemopexinless proteoform. Considering that our interpretation relied heavily on the positions of the immune-reactive bands in relation to the protein markers, we merged the obtained images from both Western Blots to create an unbiased false-colour overlay of our results **(** **Figure 3C** **)**. More specifically, the MMP-9 Western blot (green colour) was different from the anti-PEX9 blot (in opposing purple colour). At 57 kDa, only green immune-reactive bands without hemopexin domain were visible. Protein bands at/below 25 kDa matched with intact and proteolytic fragments of the hemopexin domain of MMP-9 (34). *Via* the AMC-method with our antibody N1B8, we identified citrullinated MMP-9 proteoforms at 57 kDa and 25 kDa on Western Blot **(** **Figure 3D**, red arrowheads b and c). With the latter method and reagents, we were able to improve considerably our original findings **(** **Supplementary Figure 2** **)** and achieved a drastic increase in detection sensitivity. Multiple gelatin-binding proteoforms were citrullinated and all RA synovial fluids contained citrullinated hemopexin-less MMP-9. This 57 kDa proteoform was also detectable in low amount in the synovial fluid from a control patient with traumatic arthritis. As a control, we performed gelatin-zymography analysis on the same samples **(** **Figure 3E** **)** and corroborated the findings about MMP-2 and MMP-9 proteoforms, observed in **Figure 1A**.

## Discussion

Recently, we documented efficient *in vitro* citrullination of MMP-9 and MMP-1, whereas MMP-2 and MMP-8 were barely citrullinated (16). Our *ex vivo* data reinforced information on RA and OA. It becomes also clear from biomarker searches and proteomic profiling (including MMPs) analysis that OA may be regarded as a pericellular matrix disease with major contributions of chondrocytes, whereas RA is dominated by infiltrating neutrophils and their effector molecules (35). In a recent overview about proteomic profiling of arthritic synovial fluids, the analysis of various MMPs was placed in such context (36). MMP-8 and MMP-9 possess relatively good specificity for RA (in comparison with OA). To enhance selectivity and thanks to the possibilities of multiplexing the use of biomarker combinations is gradually gaining importance. However, such types of analysis remain expensive and labour-intensive. Therefore, we advocate alternative approaches, such as the combined analysis of covalently linked molecules or the combination of a specific analyte in association with a disease-specific PTM. As example of the former, the complex between NGAL and MMP-9 constitutes a better diagnostic for inflammatory bowel disease than MMP-9 as a single analyte (29). In the present study, we provided a first example of the second approach: the analysis of a specific analyte (MMP-9) together with a PTM specific for RA, namely citrullination (2, 3).

Detection of citrullinated MMP-9 proteoforms with commercial reagents was originally a challenge. Both immunohistochemical and orthogonal methods for detecting citrullination suffer from technical difficulties (37), because citrullination yields an atomically small (0.98 Da) modification. False positive detection of citrullination is a common problem caused by cross-reactive antibodies and inaccurate proteomic analyses (24, 37). The more reliable method to detect modified citrullines by Western blot analysis bypasses this technical issue by modification of peptidylcitrulline to a larger chemical group (238 Da) (23). Therefore, we chose to produce monoclonal antibodies that detect this chemical modification and focused on the quality of these new monoclonal antibodies **(** **Figure 2** **)**.

The presence of citrullinated MMP-9 proteoforms might be intrinsically linked to RA pathology, rather than general synovial inflammation as also observed in OA or caused by trauma. As we were limited by the number of patients included in this study, further research is required to support our hypothesis and to unravel the molecular mechanisms driving MMP citrullination in arthritic diseases.

In conclusion, the detection of net gelatinolytic activity and the presence of the 85 kDa activation form of MMP-9 are valuable parameters for better understanding the pathophysiology and disease progression of RA. Citrullinated proteoforms of MMP-9, in particular the hemopexin-less MMP-9, were detected in RA synovial fluid and offer a new perspective into RA pathology and the role of posttranslationally modified gelatinases in this disease.

## Data Availability Statement

The original contributions presented in the study are included in the article/**Supplementary Material**. Further inquiries can be directed to the corresponding author.

## Ethics Statement

The studies involving human participants were reviewed and approved by Ethics Committee of UZ Leuven. The patients/participants provided their written informed consent to participate in this study.

## Author Contributions

BG collected most samples from patient cohorts, referred to the Rheumatology Clinic of the University Hospital Pellenberg between 1991 and 1999. IVA, RC, EM, and PF performed various analyses on these samples. These analyses included the measurements of cytokines, chemokines and MMPs. BG and GO coordinated the research and wrote the first draft of the manuscript. KY, LB, and EM performed all analyses after 2016. RJ, NB, IR, and PP produced the monoclonal antibodies. All authors contributed to the article and approved the submitted version.

## Funding

This work was supported by research grants of the Foundation for Scientific Research of Flanders (FWO-Vlaanderen) and of KU Leuven (C1 project C14/16/010). LB received a doctoral fellowship from the FWO-Vlaanderen and JV is postdoctoral fellow of the FWO-Vlaanderen.

## Conflict of Interest

The authors declare that the research was conducted in the absence of any commercial or financial relationships that could be construed as a potential conflict of interest.

## Publisher’s Note

All claims expressed in this article are solely those of the authors and do not necessarily represent those of their affiliated organizations, or those of the publisher, the editors and the reviewers. Any product that may be evaluated in this article, or claim that may be made by its manufacturer, is not guaranteed or endorsed by the publisher.
