# Supplementary material for: Proteoform Analysis of Matrix Metalloproteinase-9/Gelatinase B and Discovery of Its Citrullination in Rheumatoid Arthritis Synovial Fluids
Source: Front Immunol. 2021 Nov 29;12:763832. doi: 10.3389/fimmu.2021.763832 (PMC8667337; doi:10.3389/fimmu.2021.763832)
Supplement: Supplementary file 1 [file Presentation_1.pptx]

## Slide 1
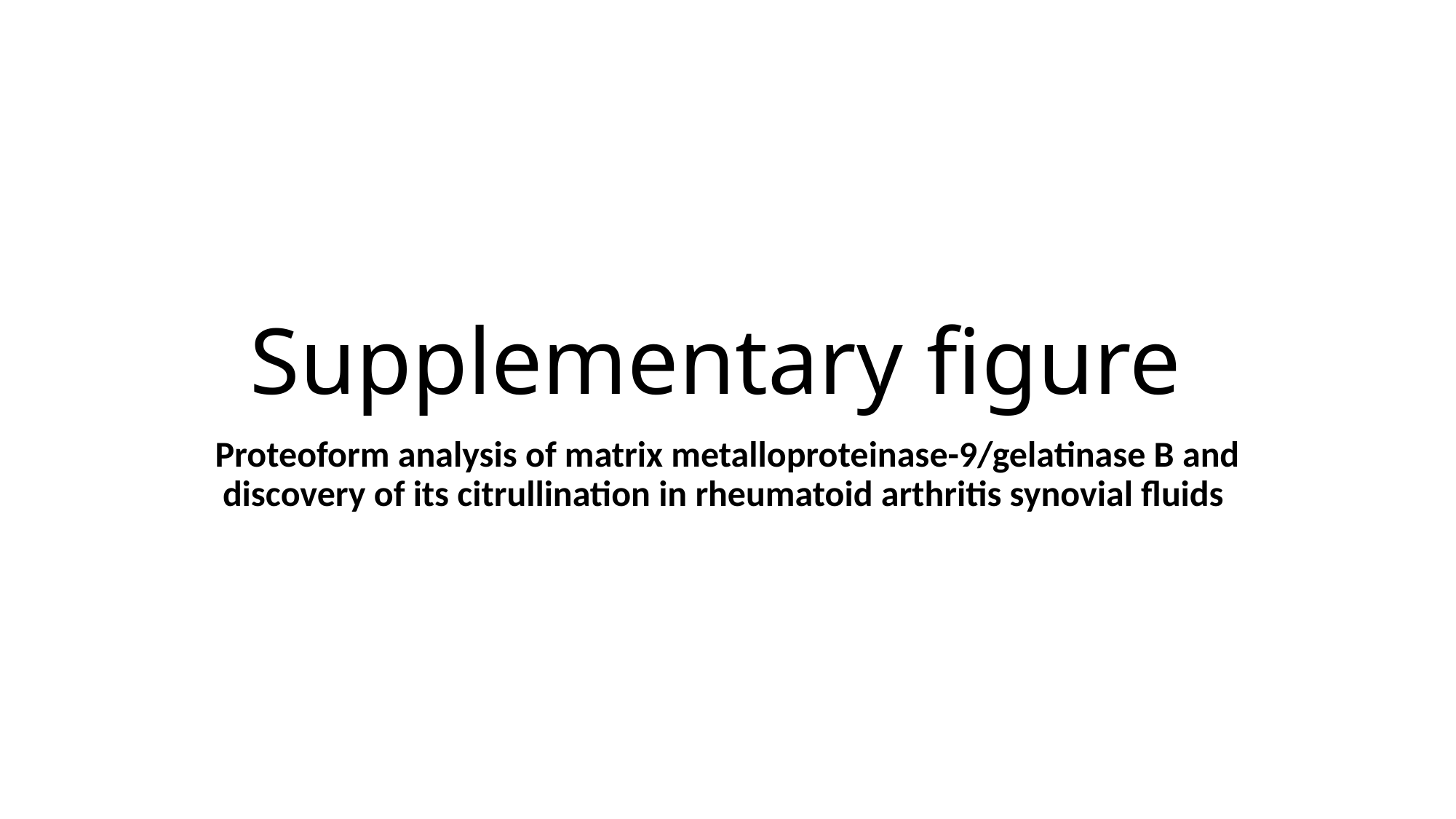

# Supplementary figure
Proteoform analysis of matrix metalloproteinase-9/gelatinase B and discovery of its citrullination in rheumatoid arthritis synovial fluids

## Slide 2
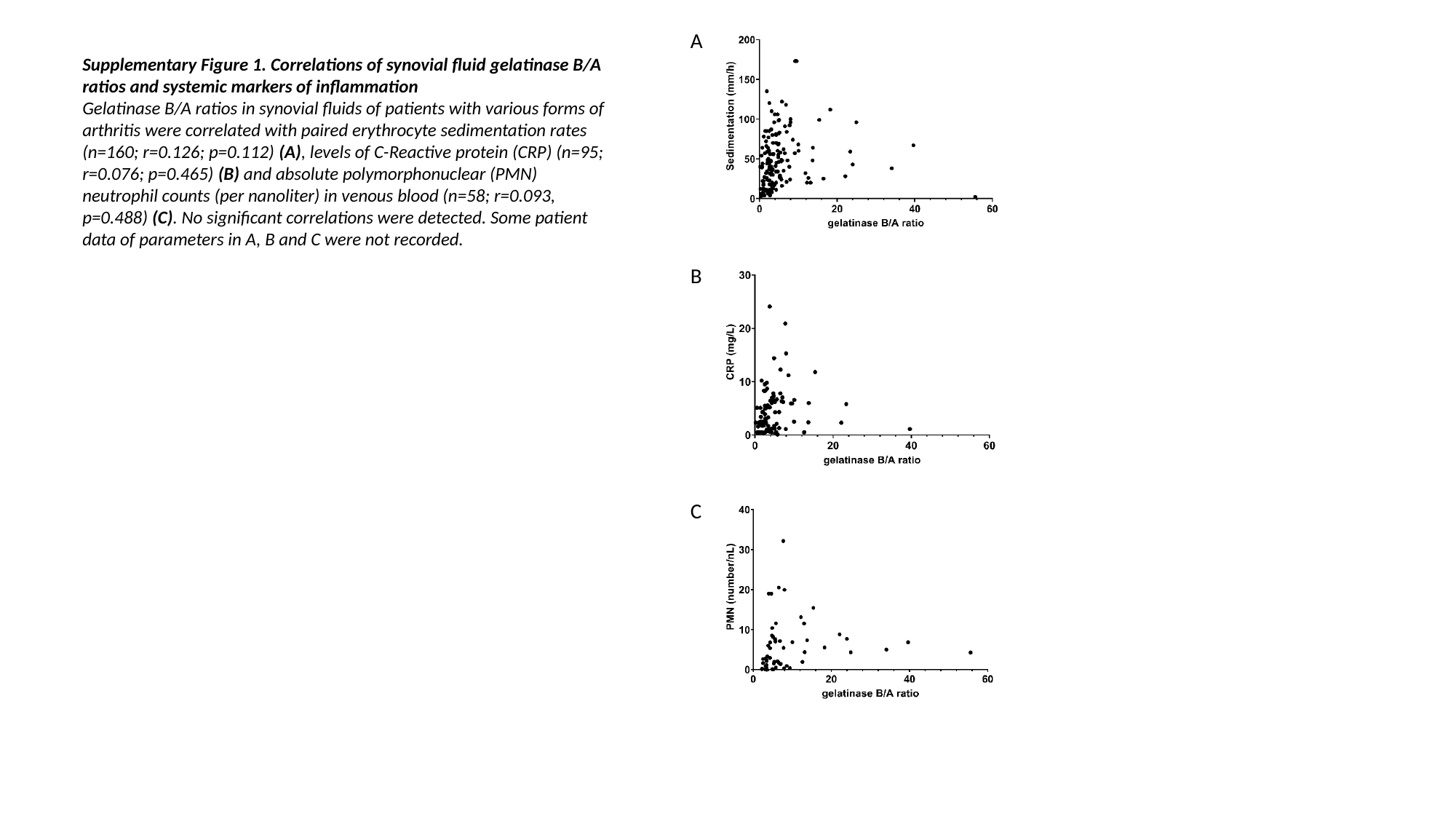

A
Supplementary Figure 1. Correlations of synovial fluid gelatinase B/A ratios and systemic markers of inflammation
Gelatinase B/A ratios in synovial fluids of patients with various forms of arthritis were correlated with paired erythrocyte sedimentation rates (n=160; r=0.126; p=0.112) (A), levels of C-Reactive protein (CRP) (n=95; r=0.076; p=0.465) (B) and absolute polymorphonuclear (PMN) neutrophil counts (per nanoliter) in venous blood (n=58; r=0.093, p=0.488) (C). No significant correlations were detected. Some patient data of parameters in A, B and C were not recorded.
B
C

## Slide 3
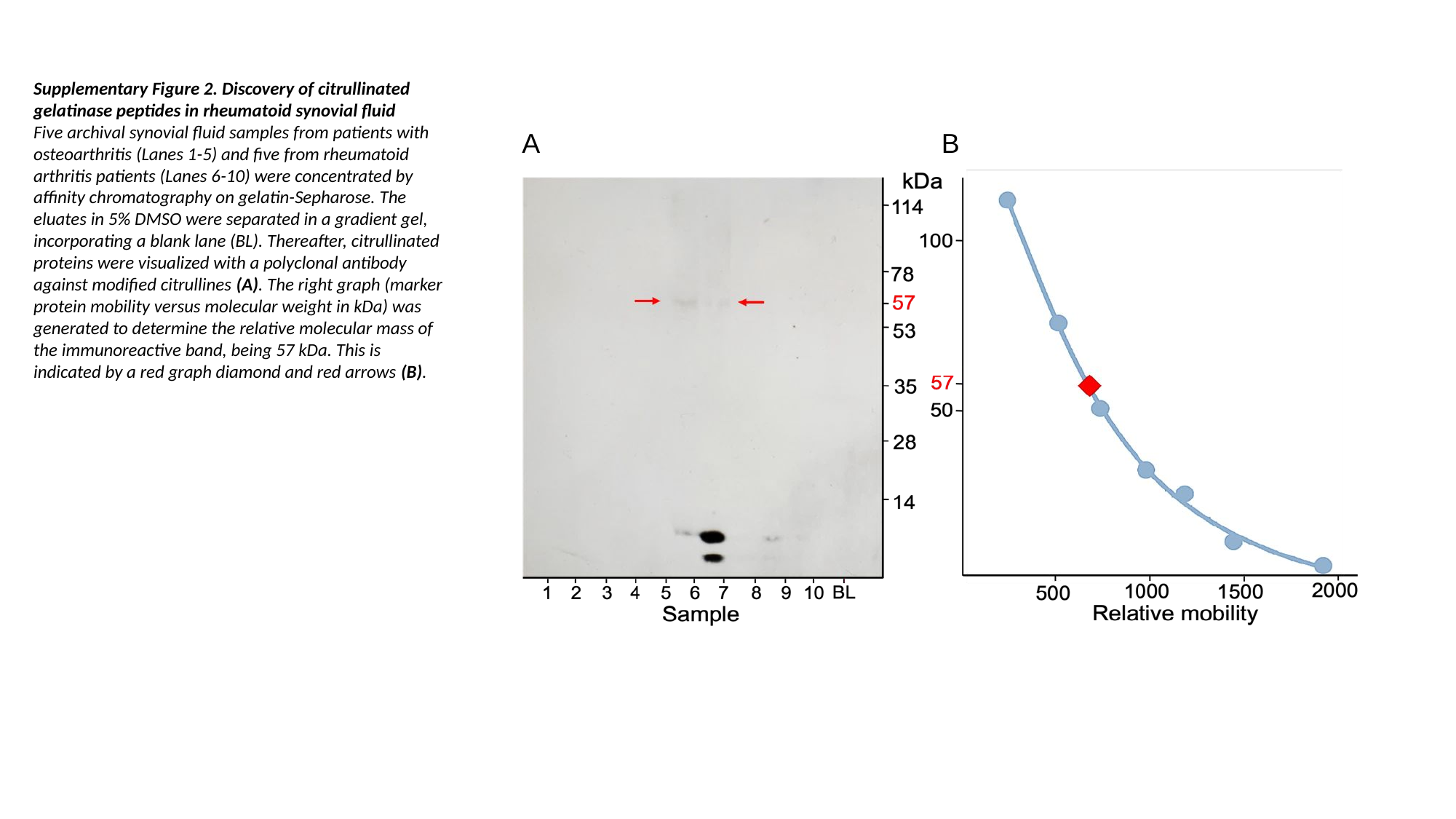

Supplementary Figure 2. Discovery of citrullinated gelatinase peptides in rheumatoid synovial fluid
Five archival synovial fluid samples from patients with osteoarthritis (Lanes 1-5) and five from rheumatoid arthritis patients (Lanes 6-10) were concentrated by affinity chromatography on gelatin-Sepharose. The eluates in 5% DMSO were separated in a gradient gel, incorporating a blank lane (BL). Thereafter, citrullinated proteins were visualized with a polyclonal antibody against modified citrullines (A). The right graph (marker protein mobility versus molecular weight in kDa) was generated to determine the relative molecular mass of the immunoreactive band, being 57 kDa. This is indicated by a red graph diamond and red arrows (B).
A
B
